# Vitamin B-12 Status during Pregnancy and Child’s IQ at Age 8: A Mendelian Randomization Study in the Avon Longitudinal Study of Parents and Children

**DOI:** 10.1371/journal.pone.0051084

**Published:** 2012-12-05

**Authors:** Carolina Bonilla, Debbie A. Lawlor, Amy E. Taylor, David J. Gunnell, Yoav Ben–Shlomo, Andrew R. Ness, Nicholas J. Timpson, Beate St Pourcain, Susan M. Ring, Pauline M. Emmett, A. David Smith, Helga Refsum, Craig E. Pennell, Marie-Jo Brion, George Davey Smith, Sarah J. Lewis

**Affiliations:** 1 School of Social and Community Medicine, University of Bristol, Bristol, United Kingdom; 2 MRC Centre for Causal Analyses in Translational Epidemiology, School of Social and Community Medicine, University of Bristol, Bristol, United Kingdom; 3 School of Oral and Dental Sciences, University of Bristol, Bristol, United Kingdom; 4 Department of Pharmacology, University of Oxford, Oxford, United Kingdom; 5 Department of Nutrition, Institute of Basic Medical Sciences, University of Oslo, Oslo, Norway; 6 School of Women’s and Infant’s Health, University of Western Australia, Australia; The University of Texas M. D. Anderson Cancer Center, United States of America

## Abstract

Vitamin B-12 is essential for the development and maintenance of a healthy nervous system. Brain development occurs primarily *in utero* and early infancy, but the role of maternal vitamin B-12 status during pregnancy on offspring cognitive function is unclear. In this study we assessed the effect of vitamin B-12 status in well-nourished pregnant women on the cognitive ability of their offspring in a UK birth cohort (ALSPAC). We then examined the association of SNPs in maternal genes *FUT2* (rs492602) and *TCN2* (rs1801198, rs9606756) that are related to plasma vitamin B-12, with offspring IQ. Observationally, there was a positive association between maternal vitamin B-12 intake and child’s IQ that was markedly attenuated after adjustment for potential confounders (mean difference in offspring IQ score per doubling of maternal B-12 intake, before adjustment: 2.0 (95% CI 1.3, 2.8); after adjustment: 0.7 (95% CI −0.04, 1.4)). Maternal *FUT2* was weakly associated with offspring IQ: mean difference in IQ per allele was 0.9 (95% CI 0.1, 1.6). The expected effect of maternal vitamin B-12 on offspring IQ, given the relationships between SNPs and vitamin B-12, and SNPs and IQ was consistent with the observational result. Our findings suggest that maternal vitamin B-12 may not have an important effect on offspring cognitive ability. However, further examination of this issue is warranted.

## Introduction

Vitamin B-12 (cobalamin) is an essential nutrient that plays a key role in DNA synthesis, S-adenosylmethionine-dependent methylation reactions, red blood cell formation and maintenance of a healthy nervous system [1]. It is not produced by the human body and can be obtained only through intake of foods of animal origin (such as meat, eggs, dairy products) or supplementation. Vitamin B-12 deficiency arises because of low dietary intake or, more frequently, because of failure to absorb it, and leads to elevated methylmalonic acid (MMA) and homocysteine (Hcy) concentration. High circulating homocysteine, which is converted to methionine within the one-carbon cycle, has been linked to early abortion, pregnancy complications, haematological abnormalities, as well as impaired growth and poor cognitive development in infancy and childhood [2], [3].

Vitamin B-12 concentration in breast milk and vitamin B-12 status of the infant are associated with vitamin B-12 concentration in maternal blood [4]. Because of the transfer from mother to offspring of vitamin B-12 during pregnancy and lactation, maternal requirements during these periods are increased and deficiency can occur [5]. Despite the importance of vitamin B-12 to neuronal development and the dependency of the developing foetus and infant on maternal vitamin B-12 status, it is unknown whether variation in maternal pregnancy vitamin B-12, across the normal range, influences offspring neurodevelopment. One study in India [6] and one in Mexico [7] suggested a positive association of maternal vitamin B-12 with offspring cognitive ability, but a second study in India found no such association [8]. A problem with observational studies is that residual confounding may explain any observed association [9]. One way to avoid problems of residual confounding in observational epidemiology is to use genetic variants that are known to be reliably related to the modifiable risk factor of interest (here maternal vitamin B-12) as proxies in order to determine the causal effect of the risk factor [9], [10].

Recent genome-wide association studies (GWAS) have shown that variants of the alpha (1, 2) fucosyltransferase 2 (*FUT2)* gene are associated with plasma concentrations of vitamin B-12 among individuals of European ancestry [11], [12]. Additionally, non-synonymous polymorphisms in the *TCN2* gene (rs1801198, 776C>G, P259R; rs9606756, 67A>G, I23V), that codes for the transcobalamin II protein, have been associated with differences in the holotranscobalamin and total transcobalamin fractions of vitamin B-12 and total Hcy concentration [3], [13]–[16], although their effects are not fully established [16].

**Figure 1:**
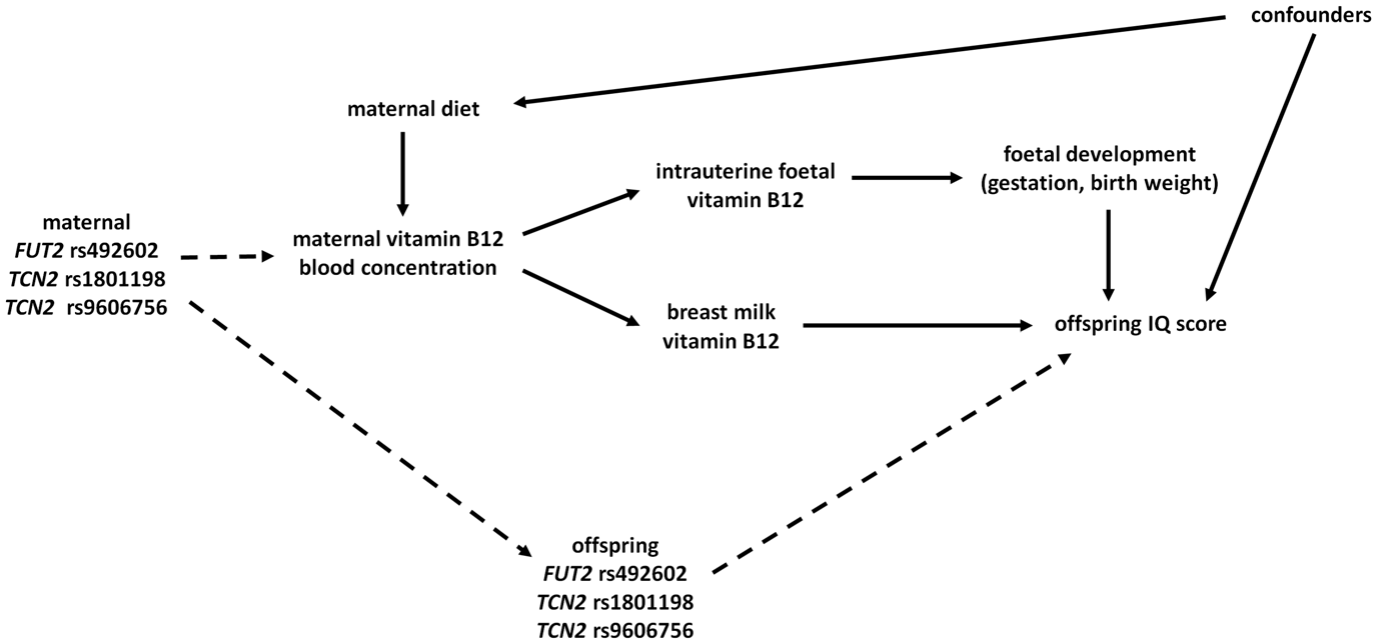
Diagram showing the framework of the Mendelian randomization approach used in this study.

The aim of this study was to examine the association of maternal dietary intake of vitamin B-12 and also of SNPs in *FUT2* and *TCN2* with offspring IQ assessed at age 8 in a population of European origin. Figure 1 shows the framework for the hypothesis that we have examined here concerning the links between maternal vitamin B-12 during pregnancy with offspring IQ.

## Materials and Methods

### Ethics Statement

Ethical approval for the study was obtained from the ALSPAC Law and Ethics Committee (IRB# 00003312) and the Local Research Ethics Committees (Bristol and Weston, Southmead, and Frenchay Health Authorities). Written informed consent was obtained from all participants in the study. Parents provided written informed consent for their child.

### Study Population

The Avon Longitudinal Study of Parents and Children (ALSPAC) is a population-based prospective cohort study investigating factors that affect the health and development of children and their parents. The study methods are described in detail elsewhere [17]–[18] (http://www.alspac.bris.ac.uk). Briefly, pregnant women living around Bristol, England, who had an expected date of delivery between April 1991 and December 1992 were eligible, and of these over 14,000 were enrolled in the study. Extensive data have been collected from the mothers and their offspring from pregnancy onwards by questionnaire, abstraction from medical notes, record linkage and by attendance at research clinics. The representativeness of the ALSPAC cohort with respect to the population living in Avon county and the whole of Great Britain in 1991 has been assessed and is reported on the ALSPAC website (http://www.bristol.ac.uk/alspac/researchers/resources-available/cohort/represent/).

**Figure 2:**
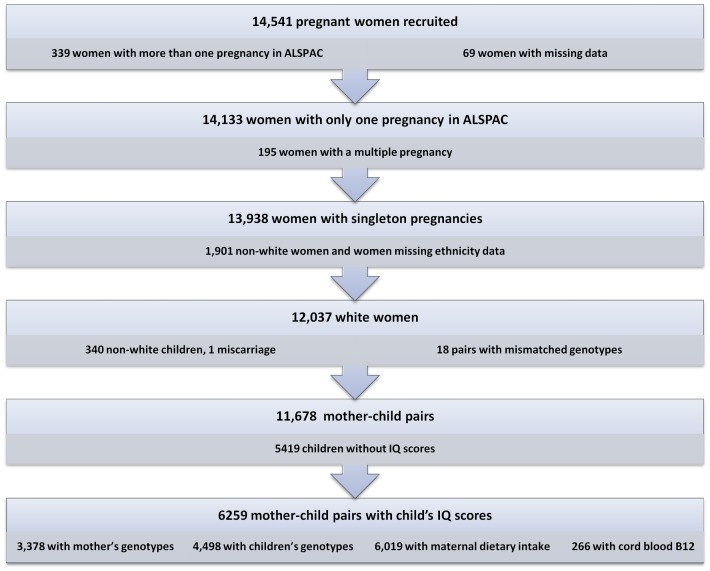
Flow-diagram of participants in the study, and reasons for exclusions.

In this study, we only included white mother-offspring pairs from singleton pregnancies (Figure 2). Numbers in each analysis varied according to the exposure and outcome being examined. Population characteristics are shown in Table 1.

**Table 1:** Characteristics of the study population.

continuous variables	mean	SD	N
mother’s age at delivery (years)	28.2	4.9	11678
gestation at delivery (weeks)	39.5	1.7	11678
birth-weight (grams)	3433.1	532.9	11530
child’s age at testing (months)	103.4	3.3	6371
full scale IQ	104.4	16.4	6259
categorical variables	no.	%	N
**mother’s education**			11144
less than O level	3290	29.5	
O level	3913	35.1	
more than O level	3941	35.4	
**mother’s social class**			9160
manual	1815	19.8	
non-manual	7345	80.2	
**child’s sex**			11677
male	6027	51.6	
female	5650	48.4	
**parity**			11169
no children	5017	44.9	
1 child	3948	35.4	
2 children	1581	14.2	
3 or more children	623	5.6	
**breastfeeding**			9684
never	2531	26.1	
<3 months	2208	22.8	
3–5 months	1607	16.6	
6+ months	3338	34.5	
**any infection this pregnancy**			10388
no	8079	77.8	
yes	2309	22.2	
**ever smoked**			11213
no	5589	49.8	
yes	5624	50.2	
**alcohol consumption**			
*before this pregnancy*			11220
never	787	7.0	
<1 glass per week	4229	37.7	
≥1 glass per week	4929	43.9	
≥1 glass per day	1275	11.4	
*months 1–3 this pregnancy*			11192
never	5000	44.7	
<1 glass per week	4412	39.4	
≥1 glass per week	1582	14.1	
≥1 glass per day	198	1.8	
**folate supplementation in pregnancy**			11529
no	8232	71.4	
yes	3297	28.6	

### Assessment of Offspring Cognitive Function

Cognitive testing was carried out by trained psychologists during a clinic visit when the children were aged 8 years using a shortened version of the Wechsler Intelligence Scale for Children (WISC-III) consisting of alternate items only (except the coding task) [19]. An overall age-scaled intelligence quotient (IQ) according to WISC manual guidelines was derived from this assessment for each child who completed the test [20]. There were 6259 children with an IQ score available for this study.

### Assessment of Cord Blood Vitamin B-12 Concentration

A 10% sample of the ALSPAC cohort, known as the Children in Focus (CiF) group, attended clinics at the University of Bristol at various time intervals between 4 and 61 months of age. The CiF group and an additional similarly selected `control’ group were chosen at random from the last six months of ALSPAC recruitment. Excluded were mothers who had moved out of the area or were lost to follow up. From these groups, five hundred children (250 girls, 250 boys) were selected to contribute a cord blood sample. Only 331 had suitable cord samples. Out of these 331, a total of 310 (149 girls, 161 boys) had plasma concentrations of vitamin B-12 determined using a colistin sulfate-resistant strain of *Lactobacillus leichmanii* [21], [22]. The coefficient of variation for vitamin B-12 was 7% based on control samples run with every assay. The association of cord blood vitamin B-12 with vitamin B-12-related genetic variants was assessed as part of the Mendelian randomization analysis. This sub-sample of children with cord blood vitamin B-12 measures was included in the larger ALSPAC sample that was used to examine the association between vitamin B-12 related genetic variants and IQ.

### Assessment of Maternal Vitamin B-12 Dietary Intake

Maternal vitamin B-12 intake was assessed in the whole cohort at 32 weeks of pregnancy using a food frequency questionnaire (FFQ). The FFQ included questions about the weekly frequency of consumption of 43 food groups and food items [23]. The foods used to determine the nutrient content of each food group were chosen to represent the type of foods normally eaten by women in this area of the UK and nutrient content of these foods was obtained from British food composition tables [24]. The FFQ was used to calculate approximate energy and nutrient intakes by multiplying the weekly frequency of consumption of each food/food group by the nutrient content (for this specific study vitamin B-12) of a standard portion of that food (the portion sizes were suitable for women of childbearing age). There were 10851 women with dietary intake information.

### Potential Confounders and Mediators

We tested for the association of potential confounders with offspring IQ, maternal dietary vitamin B-12 intake and genotypes of vitamin B-12 related variants. Information on maternal education, social class, age at delivery, parity, smoking, alcohol consumption and folate supplementation during pregnancy was obtained by questionnaires completed by the mothers at approximately 18 and 32 weeks of pregnancy. Occurrence of any infection during pregnancy was abstracted from medical records. Child’s date of birth, birth-weight and sex were obtained from birth records. In the observational analyses of maternal dietary vitamin B-12 in pregnancy, we also examined possible mediation by gestational age, birth-weight and breastfeeding (obtained from repeat postnatal maternal completed questionnaires and derived as no breastfeeding, breastfeeding for <3 months, for 3 to 5 months, for ≥6 months).

### Genetic Variants

DNA was extracted as described previously [25]. Genotyping of rs492602 and rs1801198 was undertaken by KBioscience Ltd. (www.kbioscience.co.uk), which uses a proprietary competitive allele specific PCR system (KASPar) for SNP analysis. rs9606756 was not included in the set of SNPs genotyped at Kbioscience and was therefore imputed from a GWAS performed in ALSPAC mothers and children using Markov Chain Haplotyping software (MACH v.1.0) [26] and phased haplotype data from individuals of European ancestry (CEU) (Hapmap release 22, Phase II NCBI B36, dbSNP 126) based on a cleaned dataset of 8,340 mothers and 8,365 children and 464,311 autosomal SNPs. Genotypic dosages were derived from genome-wide data and rounded for analysis. As an indication of imputation quality, rs9606756 genotypes from imputation had an R^2^ value exceeding 0.99. Numbers of mother and children pairs with genotypic data available are shown in Figure 2.

Haplotypes consisting of both *TCN2* SNPs were evaluated as potential instrumental variables that may explain better than genotypes part of the variation in circulating vitamin B-12.

The top 10 principal components that reflect the population’s genetic structure were estimated according to Price et al. [27]. Adjustment for population stratification was accomplished by including as covariates in the analyses of the ALSPAC cohort all principal components that were associated with the phenotype and the genetic variants. For the mothers, these were principal components 1, 4, 6, 8 and 9, whereas for the children, principal components 3, 4, 5, 6 and 10 were included.

### Statistical Analysis

Maternal dietary vitamin B-12 intake and cord vitamin B-12 measures were log transformed because they were positively skewed. We investigated the association of potential confounding factors with maternal vitamin B-12 intake, offspring IQ, and maternal and offspring genotype using linear regression and chi square tests. Linear regression was also used in the analysis of the association of maternal dietary intake, cord blood vitamin B-12 and genotypes with offspring IQ.

#### Observational analysis

When examining maternal dietary intake and offspring IQ, we adjusted for child’s age and sex, and maternal energy intake (model 1), additional potential confounders (maternal education, social class, age at delivery, parity, infection in pregnancy, smoking, alcohol consumption before and during pregnancy, and folate supplementation; model 2), and finally potential mediators (gestational age, birth-weight and breastfeeding duration; model 3) (Table 2). The association of cord blood vitamin B-12 and offspring IQ was similarly adjusted for covariates and mediators. In these analyses, we log transformed vitamin B-12 to base 2 so that results are interpreted as a mean difference in IQ per doubling of vitamin B-12 intake or status.

**Table 2:** Association of maternal dietary vitamin B-12 intake during pregnancy with offspring IQ at age 8. N = 4787 with complete data on exposure, outcome and covariables in any model.

	mean difference in child IQ per doubling of maternal vitamin B-12 intake	95% CI	p-value
Model 1	2.0	1.3, 2.8	<0.001
Model 2	0.7	−0.04, 1.4	0.06
Model 3	0.5	−0.2, 1.2	0.15

Model 1: Adjusted for offspring sex and age at time of IQ assessment, and maternal energy intake.

Model 2: As model 1 plus additional adjustment for potential confounding by maternal education, social class, age at delivery, parity, infection in pregnancy, ever smoked, alcohol consumption before and during pregnancy, and folate supplementation.

Model 3: As model 2 plus additional adjustment for potential mediation by gestational length, birth-weight and breastfeeding duration.

#### Genetic analysis

Maternal and offspring genotypes were coded 0, 1, 2, based on the number of putative vitamin B-12 increasing alleles, and were checked for deviation from Hardy-Weinberg equilibrium using the genhw function implemented in the program Stata 11.0 (Stata Corporation, College Station, Texas). The association of maternal genotype with offspring IQ was adjusted for child’s genotype and population stratification. As offspring genotype may influence cognitive ability *per se*, we adjusted for offspring genotype to estimate the effect of maternal vitamin B-12 status during pregnancy on offspring IQ. We used an additive genetic model taking as baseline the genotype associated with the lowest vitamin B-12 and/or highest tHcy concentrations (TT for *FUT2* rs492602, GG for *TCN2* rs1801198 and AA for *TCN2* rs9606756). We also tested a recessive model for *FUT2* and *TCN2* rs9606756 as suggested elsewhere [11], [15].

The calculation of linkage disequilibrium (LD), haplotype construction and haplotype association with cord blood vitamin B-12 and offspring IQ were carried out with the program PLINK v1.07 [28].

We examined whether there was statistical evidence for an interaction between the genetic variants and maternal dietary intake in their association with offspring IQ, as this relationship may be different depending on intake. For example, poor absorption could be more relevant for individuals with reduced vitamin B-12 intake. In these analyses we stratified maternal-offspring pairs by whether the maternal intake was below the recommended daily amount (RDA) for vitamin B-12 for pregnant women (2.6 µg) or at or above this value.

Unless specified all analyses described above were conducted with the statistical package Stata 11.0.

## Results

Mean IQ among the 6259 ALSPAC children (49.9% male) with a cognitive measurement was 104.4 (SD, 16.4) and they were assessed at a mean age of 8 years 7 mo (SD, 3.3 mo). The numbers included in different analyses of association with vitamin B12-associated SNPs are shown in Figure 2. The representativeness of the ALSPAC subsets used in the analyses of IQ and cord blood vitamin B12 is examined in Tables S1 and S2.

### Dietary Maternal Vitamin B-12 Intake, Cord Blood Vitamin B-12 and Offspring IQ

Mother’s dietary intake of vitamin B-12 during pregnancy was associated with offspring IQ in simple age, sex and energy intake adjusted analyses (model 1), with offspring IQ being on average 2.0 (95%CI 1.3, 2.8; p<0.001) points greater per doubling of maternal dietary vitamin B-12 intake (Table 2). Subsequent adjustment for potential confounders attenuated the association by 65% (model 2), such that in this model offspring IQ was on average 0.7 (95%CI −0.04, 1.4; p = 0.06) points greater per doubling of maternal vitamin B-12. There was little further change upon adjustment for potential mediators. Cord blood vitamin B-12 was also positively associated, by a similar magnitude, with IQ (mean age and sex adjusted difference in offspring IQ per doubling of cord vitamin B-12∶2.9, 95% CI −0.1, 5.8, p = 0.06). Additional adjustment by confounders and mediators (mean difference in offspring IQ per doubling of cord vitamin B-12∶1.3, 95% CI −1.5, 4.1, p = 0.36) in the subsample of 222 children with these data reduced the effect by ∼55%. Because of the small sample size available for these analyses, we had only ∼50% power to detect a 2 point difference in offspring IQ.

There was no strong evidence that maternal vitamin B-12 dietary intake was associated with cord blood vitamin B-12 (ratio of geometric means per doubling of maternal intake: 1.1, 95% CI 1.0, 1.2, p = 0.10), again due to small numbers we had limited power to detect an association between the two.

### Vitamin B-12 Status Related SNPs, Maternal Dietary Intake of Vitamin B-12 and Cord Blood Vitamin B-12

Neither of the maternal SNPs was associated with maternal dietary intake of vitamin B-12 during pregnancy in a linear model, although *TCN2* rs1801198 heterozygotes showed higher vitamin B-12 intake than either homozygote group (p = 0.02). All three maternal SNPs showed a similar magnitude of effect on cord blood vitamin B-12 concentration, but statistical evidence for the effect of the *FUT2* SNP and *TCN2* rs9606756 was absent in this small sample (Table 3). Analysis of offspring genotypes and cord blood vitamin B-12 revealed a strong association with the *FUT2* variant but no association with *TCN2* rs1801198 and a marginal effect of *TCN2* rs9606756 (Table S3). Maternal rs9606756/rs1801198 haplotypes carrying at least one putative vitamin B-12 increasing allele showed higher cord blood vitamin B-12 compared to the haplotype carrying both alleles previously associated with elevated Hcy and lower vitamin B-12 concentrations (AG, i.e. encoding aminoacids Isoleucine (I) at locus rs9606756 and Arginine (R) at locus rs1801198). However, there was only evidence of an effect when comparing the AG haplotype with all others (ratio of geometric means per AG copy 0.9; 95%CI 0.8, 1.0) (Table 4). Offspring haplotypes were not associated with cord blood vitamin B-12 (data not shown).

**Table 3:** Association of maternal genotype at SNPs related to vitamin B-12 metabolism with maternal dietary intake at 32 weeks pregnant and vitamin B-12 cord blood concentration.

SNP	genotype	N^a^	median (IQR) of maternal vitaminB-12 dietary intake (µg/day)	N	median (IQR) of vitamin B-12 cord blood (pmol/L)
*FUT2* rs492602	TT	1642	4.2 (3.0, 6.1)	62	291 (183, 397)
	TC	3187	4.2 (3.0, 6.1)	128	300 (196, 424)
	CC	1648	4.3 (3.0, 6.2)	50	294 (214, 534)
	ratio of geometric means perC allele (95% CI)	6477	1.00 (0.98, 1.02)	240	1.08 (0.97, 1.19)
	p-value^a^		0.82/0.96		0.15/0.31
*TCN2* rs1801198	GG	1276	4.1 (3.0, 5.9)	41	276 (173, 369)
	CG	3221	4.3 (3.2, 6.2)	125	279 (196, 417)
	CC	2002	4.1 (3.0, 6.1)	68	318 (226, 436)
	ratio of geometric meansper C allele (95% CI)	6499	1.01 (0.99, 1.03)	234	1.13 (1.02–1.25)
	p-value^a^		0.44/0.02		0.02/0.07
*TCN2* rs9606756	AA	5483	4.3 (3.1, 6.2)	193	288 (205, 397)
	AG	1650	4.3 (3.0, 6.1)	58	298 (206, 484)
	GG	120	4.0 (2.9, 6.1)	2	238 (82, 394)
	ratio of geometric meansper G allele (95% CI)	7253	0.99 (0.96, 1.01)	253	1.03 (0.89, 1.18)
	p-value^a^		0.23/0.46		0.73/0.31

a N reflects the number of mothers with genotype and dietary intake or offspring cord blood vitamin B-12 data, regardless of whether there was offspring IQ data available for them as well.

b additive test/any effect test.

**Table 4:** Association of maternal *TCN2* haplotypes with vitamin B-12 cord blood levels and offspring IQ at age 8.

rs9606756-rs1801198 haplotype	haplotype frequency	cord blood geometricmean (95% CI)	p-value^a^	IQ mean (95% CI)	p-value^a^
AC	0.46	298 (274, 324)	0.11	104.4 (103.7, 105.1)	0.35
AG	0.41	278 (255, 304)	0.02	104.1 (103.4, 104.7)	0.03
GC	0.10	317 (257, 391)	0.23	104.7 (103.4, 105.9)	0.38
GG	0.03	279 (78, 995)	0.85	108.6 (105.4, 111.8)	0.01
**p-value** b		0.14		0.01	
**N** c	5864	202		3429	

a p-value for testing the effect of each haplotype vs all others.

b p-value for the omnibus test that compares the alternate model (each haplotype having a unique effect) vs the null model (no haplotypes having any effect).

c N corresponds to the number of mothers with available genotype data for both SNPs (N = 5866), whose children had vitamin B-12 measured in cord blood (N = 202), or whose children had been IQ tested (N = 3429).

### Maternal and Offspring Genotype at Vitamin B-12 Related SNPs and Offspring IQ

Unadjusted associations of each maternal genotype with offspring IQ in the ALSPAC cohort indicated that maternal *FUT2* rs492602 SNP was associated with child’s IQ, such that each additional C allele (i.e. the allele associated with higher plasma vitamin-B12) led to an increase in IQ of 0.05SD (p = 0.02) (Table 5). Under a recessive model (TT/TC vs. CC) [11], the mean difference in IQ between genotype groups was 0.07SD (p = 0.07). Mean difference in IQ was 3.7 (95% CI 0.02, 7.4; p = 0.05) IQ points (0.24SD) if a recessive pattern of inheritance was assumed for *TCN2* rs9606756 [15]. Adjustment for offspring genotype strengthened the association of all SNPs with offspring IQ whilst additional adjustment for population stratification did not lead to a major change in effects (Table S4). *TCN2* maternal haplotype analysis uncovered an association of haplotype AG with lower IQ scores in relation to all other haplotypes (mean difference in IQ per AG copy -0.9; 95%CI -1.7, -0.1) (Table 4). On the other hand, the rare haplotype GG (i.e. encoding aminoacids Valine (V) at locus rs9606756 and Arginine (R) at locus rs1801198) was associated with a considerable increase in IQ scores (mean difference in IQ per GG copy 4.2; 95%CI 1.2, 7.2).

**Table 5:** Association of maternal genotype at SNPs related to vitamin B-12 metabolism with offspring IQ at age 8.

SNP	genotype	N	IQ mean (SD)
*FUT2* rs492602	TT	1009	103.3 (16.8)
	TC	1940	104.2 (16.1)
	CC	1012	105.0 (16.7)
	mean difference in child IQ per Callele^a^(95% CI)	3961	0.86 (0.14, 1.58)
	p-value		0.02
*TCN2* rs1801198	GG	784	103.9 (16.6)
	CG	1978	104.1 (16.0)
	CC	1208	104.6 (17.0)
	mean difference in child IQ per Callele^a^ (95% CI)	3970	0.35 (−0.38, 1.08)
	p-value		0.35
*TCN2* rs9606756	AA	3673	104.5 (16.1)
	AG	1038	104.7 (17.0)
	GG	74	108.0 (14.3)
	mean difference in child IQ per Gallele^a^ (95% CI)	4785	0.55 (−0.44, 1.54)
	p-value		0.28

a Allele mentioned has been associated with higher vitamin B-12 or lower Hcy levels.

Offspring genotype was not associated with offspring IQ (*FUT2*: mean difference in IQ per C allele 0.06; 95%CI -0.6, 0.8; p = 0.86/*TCN2* rs1801198: mean difference in IQ per C allele 0.1; 95%CI -0.6, 0.8; p = 0.82/*TCN2* rs9606756: mean difference in IQ per G allele -0.5; 95% CI -1.6, 0.5; p = 0.32; all with adjustment for population stratification). Similarly, no effect of *TCN2* haplotypes was identified among children (data not shown).

The expected effects of maternal vitamin B-12 on offspring IQ, based on the effect of each genotype on cord blood vitamin B-12 and on child’s IQ, were 0.6, 1.1 and 1.6 additional IQ points per doubling of vitamin B-12 in cord blood when using *TCN2* rs1801198, rs9606756, and *FUT2*, respectively as proxies. All effect estimates were included within the 95% CI of the observed effect of cord blood vitamin B-12 on IQ scores (2.9; 95% CI -0.1, 5.8), and were compatible with a negative, null and largely positive association of vitamin B-12 levels with offspring IQ.

Fifteen percent of mothers consumed less than the RDA for vitamin B-12 dietary intake. The association of each genotype with offspring IQ stratified by whether the mother was taking less than the RDA for pregnant women or was taking the RDA or more is shown in Table S5. No evidence for interaction between maternal dietary intake and genotype was found.

### Confounders and Mediators

There was evidence of association between offspring IQ and 11 out of 14 (79%) of the covariables considered here (Table S6). Maternal dietary intake of vitamin B-12 was associated with the majority (7 out of 11 (64%)) of potential confounders and with two of the three mediators tested (Table S7). For the most part maternal and offspring genotypes were not associated with potential confounders or mediators (Tables S8–S13). No associations were detected with maternal and offspring rs1801198, one association each was detected with maternal rs492602, maternal and offspring rs9606756, and three associations were identified with offspring rs492602. The expected number of associations at the conventional alpha level of 0.05 was 1 (5% of 14 confounders).

### Genotypes and Haplotypes

All vitamin B-12 SNPs were in Hardy-Weinberg equilibrium in mothers and children (all p-values >0.05). Frequencies of the vitamin B-12 alleles were similar in the mothers and their offspring, and comparable to those found in the HapMap European population (CEU) (rs492602 C allele: 0.50 in ALSPAC, 0.53 in CEU; rs1801198 C allele: 0.55 in ALSPAC, 0.55 in CEU; rs9606756 G allele: 0.13 in ALSPAC, 0.10 in CEU). Linkage disequilibrium levels between both *TCN2* SNPs were also analogous in mothers and children (D’ = 0.48/0.44, R^2^ = 0.03/0.02). All four haplotypes were present in the population.

## Discussion

### Main Findings

To the best of our knowledge this is the first study to examine the impact of differential concentrations of circulating vitamin B-12 on offspring IQ through the use of records of maternal pregnancy vitamin B-12 intake, as well as vitamin B-12 related genetic variants. Whilst the majority of studies in adults and children have focused on the consequences of vitamin B-12 deficiency we have focused on variation across the normal range.

Maternal dietary vitamin B-12 intake was positively associated with offspring IQ, however, there was marked attenuation of the association with adjustment for potential confounding factors. *FUT2* and *TCN2* SNPs are known to be associated with plasma concentrations of vitamin B-12 and its metabolic markers [11], [12], [3], [13], [15] and are less likely to be associated with confounders than measurements of dietary intake. We therefore used them as proxy measures for plasma B-12 concentration to assess its causal effect on offspring IQ. In ALSPAC, child IQ was weakly associated with maternal vitamin B-12-elevating alleles at *FUT2* and *TCN2*. The effect size of this association between genotypic variation and IQ was of a magnitude expected given the relationships between genetic variation and vitamin B-12 and the relationships between vitamin B-12 and IQ observationally. Conditioning on offspring genotype made this association somewhat stronger, giving support to the hypothesis that offspring IQ score may be influenced by maternal vitamin B-12 status during the intra-uterine or perinatal period rather than reflect post-natal offspring exposure to dietary vitamin B-12.

### Findings in the Context of Existing Literature

*TCN2* rs1801198 was associated with maternal vitamin B-12 consumption, with heterozygotes showing a higher intake than both homozygotes, although this could well be a chance finding since this SNP’s putative action has not been related to intake [13]. rs1801198 is a non-synonymous polymorphism (P259R) that has been reported to decrease transcription and the cellular and plasma concentration of holo-transcobalamin, which is the biologically active fraction of vitamin B-12 delivered to tissues [13], [29]. *TCN2* rs9606756 is also a non-synonymous variant (I23V) that may facilitate the binding of cobalamin [30] and has been associated with a higher proportion of vitamin B-12 bound to transcobalamin [14], and lower plasma total Hcy [15]. Rs492602 in *FUT2* is a synonymous variant (A68A) in close LD with SNP rs601338 which causes a stop codon to arise (W154X). As a result of being homozygous for the null allele at rs601338, ∼20% of individuals of European ancestry do not express ABO antigens in secretions and epithelial cells (“non-secretors”). Non-secretor status has been associated with a reduced risk of infection with *Helicobacter pylori* and Norovirus, slow progression of HIV infection and a higher susceptibility for Crohn’s disease [31], [32]. Since *H. pylori* infection may cause malabsorption of vitamin B-12, non-secretors are hypothesized to have better absorption and hence higher plasma concentrations of vitamin B-12 than secretors [33]. Consistent with this hypothesis, we found that homozygotes for the rs492602 C allele (in strong LD with rs601338 null allele) exhibited higher cord blood vitamin B-12 than TT/TC individuals [geometric mean (95% CI), 322 (277, 374) vs 284 (263, 307), p = 0.15].

Previous studies of the effect of plasma vitamin B-12 on neurological development in children suggest that cobalamin deficiency has an adverse effect on children’s cognitive ability and motor skills. Louwman et al. [34] found that children who had been fed a macrobiotic diet in early childhood exhibited low cobalamin status and scored the lowest on a battery of psychological tests taken during adolescence. Although other studies have found conflicting results [35]. Case studies conducted in India, the US and Europe have reported developmental delay in children of vegan or vegetarian mothers who were exclusively breastfed up to 10 months old [36]. These infants not only showed very low vitamin B-12 concentrations in plasma but also ineffective haematopoiesis and degeneration of nervous tissue [37]. Neither supplementation therapy nor a change in diet managed to completely overturn the neurological impairments [36], [37]. We did not uncover any association between child’s genotype at vitamin B-12 related variants and IQ despite finding an association between offspring genotype and cord blood vitamin B-12 concentration. However, the majority of the children with cord blood vitamin B-12 measures in our study were not vitamin B-12 deficient. It is also likely that we did not have enough statistical power in our analysis to identify an effect of vitamin B-12 on IQ in children.

It has been previously shown that maternal cobalamin status is a strong predictor of vitamin B-12 in breastfed infants up to at least 6 months of age [35]. Yet, little is known about the influence that low plasma vitamin B-12 during pregnancy may have on the cognitive ability of children later in life. In Mexico, a study that assessed maternal diet during the first trimester of pregnancy and offspring neurodevelopment reported a decrease in cognition test scores among children of mothers with a deficient intake of vitamin B-12 (<2 µg/day) [7]. Two studies conducted in India, that measured vitamin B-12 concentration, reported contradictory results, possibly due to differing levels of vitamin B-12 deficiency between the populations [6], [8]. Alternatively, differing confounding structures could explain the dissimilar findings.

### Limitations

With respect to dietary data the use of a FFQ to estimate vitamin B-12 dietary intake without taking into account vitamin B-12 bioavailability can be considered a limitation. Recent work has shown that different foods can yield very different blood concentrations of vitamin B-12 [38]. Also, the FFQ is not accurate enough to use in analyses other than the description of dietary intake in a population, and therefore, association results emerging from the use of FFQ should be interpreted with caution. A further shortcoming is the fact that dietary intake was not adjusted for misreporting as we did not have enough information from participants to accurately do so. In terms of vitamin B-12 plasma measurements, we lacked maternal data to verify the association with *FUT2* and *TCN2* genotypes and undertake a formal instrumental variable analysis [10]. And we only had a small sample of cord blood measurements to confirm robust associations with the genetic variants and with IQ. Additionally, the SNPs that we have used here as proxies for the unconfounded association of maternal vitamin B-12 with offspring IQ explain only a small proportion of the variance of plasma vitamin B-12 [11], [39]. This means that large sample sizes are required when using them in more formal Mendelian randomization studies.

Whilst all SNPs showed positive associations with offspring IQ only one was statistically robust and confirmation is required from additional studies. Because we used smaller subsets of samples from the original ALSPAC study to assess associations of SNPs with cord blood vitamin B-12 and IQ, there is always the possibility of these subsets not being representative of the population under study. Although this might affect observational studies, given that genotypes are not associated with missingness it is unlikely to influence Mendelian randomization results [40], [41]. The differences in confounders between sample subsets and the overall ALSPAC sample are unlikely to affect the association between genotypes and cord blood vitamin B-12 or genotypes and IQ because, as expected, there was no association between genotypes and potential confounders, other than what may have arisen by chance due to performing a large number of tests on the data.

### Conclusions

In summary, we have found that children born to mothers with a higher dietary intake of vitamin B-12 had a slightly higher IQ than other children, and that those with mothers carrying putative vitamin B-12-increasing alleles were more likely to have a higher IQ than those born to mothers without these alleles. Nonetheless, the fact that our observational association of maternal dietary intake attenuated noticeably with adjustment for confounding factors and that the genetic associations are not very strong suggests that maternal vitamin B-12 may not have an important causal effect on offspring IQ. Larger Mendelian randomization studies are needed to evaluate this further.

## Supporting Information

Table S1 **Representativeness of the sub-sample of children with cord blood vitamin B-12 levels, and the sample used for IQ analysis, with respect to the full ALSPAC sample.**
(DOCX)

Table S2 **Minor allele frequencies of SNPs used in this study by analyzed sample.**
(DOCX)

Table S3 **Association of offspring genotype at SNPs related to vitamin B-12 metabolism with vitamin B-12 cord blood concentration.**
(DOCX)

Table S4 **Multivariable association of maternal genotype with offspring IQ at age 8.**
(DOCX)

Table S5 **Association of maternal genotype at SNPs related to vitamin B-12 metabolism with offspring IQ at age 8, stratified by maternal pregnancy recommended daily amount (RDA) of vitamin B-12.**
(DOCX)

Table S6 **Association of offspring IQ with potential covariables.**
(DOCX)

Table S7 **Association between maternal vitamin B-12 daily intake and potential covariables.**
(DOCX)

Table S8 **Association between maternal genotype at rs492602 and potential covariables.**
(DOCX)

Table S9 **Association between offspring genotype at rs492602 and potential covariables.**
(DOCX)

Table S10 **Association between maternal genotype at rs1801198 and potential covariables.**
(DOCX)

Table S11 **Association between offspring genotype at rs1801198 and potential covariables.**
(DOCX)

Table S12 **Association between maternal genotype at rs9606756 and potential covariables.**
(DOCX)

Table S13 **Association between offspring genotype at rs9606756 and potential covariables.**
(DOCX)
