# Supplementary material for: Vitamin B-12 Status during Pregnancy and Child’s IQ at Age 8: A Mendelian Randomization Study in the Avon Longitudinal Study of Parents and Children
Source: PLoS One. 2012 Dec 5;7(12):e51084. doi: 10.1371/journal.pone.0051084 (PMC3515553; doi:10.1371/journal.pone.0051084)
Supplement: Table S1 — Representativeness of the sub-sample of children with cord blood vitamin B-12 levels, and the sample used for IQ analysis, with respect to the full ALSPAC sample. (DOCX) [file pone.0051084.s001.docx]

**Table S1.** Representativeness of the sub-sample of children with cord blood vitamin B-12 levels, and the sample used for IQ analysis, with respect to the full ALSPAC sample.

| **variables** | **cord blood sub-sample** | | **IQ sample** | | **full sample** | | **p-value^a^** | **p-value^b^** |
| --- | --- | --- | --- | --- | --- | --- | --- | --- |
| **continuous** | **mean ± SD** | **N** | **mean ± SD** | **N** | **mean ± SD** | **N** |  |  |
| mother’s age at delivery (years) | 29.6 ± 4.5 | 310 | 29.2 ± 4.5 | 6259 | 28.2 ± 4.9 | 11678 | <0.001 | <0.001 |
| gestation at delivery (weeks) | 39.6 ± 1.4 | 310 | 39.5 ± 1.7 | 6259 | 39.5 ± 1.7 | 11678 | 0.30 | 1.00 |
| birth-weight (grams) | 3522.8 ± 470.3 | 306 | 3449.9 ± 523.7 | 6184 | 3433.1 ± 532.9 | 11530 | 0.004 | 0.04 |
| child’s age at testing (months) | 103.4 ± 1.9 | 272 | 103.4 ± 3.3 | 6259 | 103.4 ± 3.3 | 6371 | 1.00 | 1.00 |
|  |  |  |  |  |  |  |  |  |
| **categorical** | % | N | % | N | % | N |  |  |
| **mother’s education** |  |  |  |  |  |  | <0.001 | <0.001 |
| less than O level | 20.9 | 64 | 21.5 | 1327 | 29.5 | 3290 |  |  |
| O level | 34.2 | 105 | 35.1 | 2163 | 35.1 | 3913 |  |  |
| more than O level | 45.0 | 138 | 43.3 | 2669 | 35.4 | 3941 |  |  |
| **mother’s social class** |  |  |  |  |  |  | 0.08 | <0.001 |
| manual | 15.4 | 41 | 15.9 | 851 | 19.8 | 1815 |  |  |
| non-manual | 84.6 | 226 | 84.1 | 4506 | 80.2 | 7345 |  |  |
| **child’s sex** |  |  |  |  |  |  | 0.96 | 0.03 |
| male | 51.9 | 161 | 49.9 | 3120 | 51.6 | 6027 |  |  |
| female | 48.1 | 149 | 50.1 | 3139 | 48.4 | 5650 |  |  |
| **parity** |  |  |  |  |  |  | 0.57 | 0.001 |
| no children | 44.6 | 137 | 46.6 | 2852 | 44.9 | 5017 |  |  |
| 1 child | 38.4 | 118 | 35.9 | 2195 | 35.4 | 3948 |  |  |
| 2 children | 12.4 | 38 | 13.1 | 803 | 14.2 | 1581 |  |  |
| 3 or more children | 4.6 | 14 | 4.4 | 271 | 5.6 | 623 |  |  |
| **breastfeeding** |  |  |  |  |  |  | 0.04 | <0.001 |
| never | 19.5 | 57 | 19.9 | 1160 | 26.1 | 2531 |  |  |
| < 3 months | 21.8 | 64 | 22.8 | 1330 | 22.8 | 2208 |  |  |
| 3-5 months | 19.8 | 58 | 17.7 | 1035 | 16.6 | 1607 |  |  |
| 6+ months | 38.9 | 114 | 39.7 | 2317 | 34.5 | 3338 |  |  |
| **any infection this pregnancy** |  |  |  |  |  |  | 0.90 | 0.01 |
| no | 77.3 | 235 | 79.5 | 4769 | 77.8 | 8079 |  |  |
| yes | 22.7 | 69 | 20.5 | 1232 | 22.2 | 2309 |  |  |
| **ever smoked** |  |  |  |  |  |  | 0.04 | <0.001 |
| no | 56.0 | 173 | 55.8 | 3428 | 49.8 | 5589 |  |  |
| yes | 44.0 | 136 | 44.2 | 2713 | 50.2 | 5624 |  |  |
| **alcohol consumption** |  |  |  |  |  |  |  |  |
| *before this pregnancy* |  |  |  |  |  |  | <0.001 | <0.001 |
| never | 5.5 | 17 | 5.5 | 335 | 7.0 | 787 |  |  |
| < 1 glass per week | 32.1 | 99 | 37.3 | 2295 | 37.7 | 4229 |  |  |
| ≥ 1 glass per week | 43.5 | 134 | 45.2 | 2784 | 43.9 | 4929 |  |  |
| ≥ 1 glass per day | 18.8 | 58 | 12.0 | 738 | 11.4 | 1275 |  |  |
| *months 1-3 this pregnancy* |  |  |  |  |  |  | 0.06 | 0.21 |
| never | 40.1 | 123 | 43.6 | 2678 | 44.7 | 5000 |  |  |
| < 1 glass per week | 41.0 | 126 | 41.0 | 2523 | 39.4 | 4412 |  |  |
| ≥ 1 glass per week | 15.3 | 47 | 13.7 | 840 | 14.1 | 1582 |  |  |
| ≥ 1 glass per day | 3.6 | 11 | 1.7 | 103 | 1.8 | 198 |  |  |
| **folate supplementation in pregnancy** |  |  |  |  |  |  | 0.01 | 0.01 |
| no | 64.8 | 201 | 69.5 | 4334 | 71.4 | 8232 |  |  |
| yes | 35.2 | 109 | 30.5 | 1901 | 28.6 | 3297 |  |  |
