# Supplementary material for: Vitamin B-12 Status during Pregnancy and Child’s IQ at Age 8: A Mendelian Randomization Study in the Avon Longitudinal Study of Parents and Children
Source: PLoS One. 2012 Dec 5;7(12):e51084. doi: 10.1371/journal.pone.0051084 (PMC3515553; doi:10.1371/journal.pone.0051084)
Supplement: Table S2 — Minor allele frequencies of SNPs used in this study by analyzed sample. (DOCX) [file pone.0051084.s002.docx]

**Table S2.** Minor allele frequencies of SNPs used in this study by analyzed sample.

|  | **cord blood sub-sample** | **IQ sample** | **full sample** | **CEU** |
| --- | --- | --- | --- | --- |
| **mothers** |  |  |  |  |
| rs492602*T | 0.525 | 0.500 | 0.500 | 0.469 |
| rs1801198*G | 0.442 | 0.447 | 0.445 | 0.450 |
| rs9606756*G | 0.123 | 0.124 | 0.130 | 0.102 |
|  |  |  |  |  |
| **children** |  |  |  |  |
| rs492602*T | 0.474 | 0.503 | 0.504 | 0.469 |
| rs1801198*G | 0.434 | 0.443 | 0.446 | 0.450 |
| rs9606756*G | 0.120 | 0.125 | 0.128 | 0.102 |

CEU: HapMap European population.
