# Supplementary material for: Vitamin B-12 Status during Pregnancy and Child’s IQ at Age 8: A Mendelian Randomization Study in the Avon Longitudinal Study of Parents and Children
Source: PLoS One. 2012 Dec 5;7(12):e51084. doi: 10.1371/journal.pone.0051084 (PMC3515553; doi:10.1371/journal.pone.0051084)
Supplement: Table S3 — Association of offspring genotype at SNPs related to vitamin B-12 metabolism with vitamin B-12 cord blood concentration. (DOCX) [file pone.0051084.s003.docx]

**Table S3.** Association of offspring genotype at SNPs related to vitamin B-12 metabolism with vitamin B-12 cord blood concentration.

|  |  |  | **Vitamin B-12 cord blood (pmol/L)** |
| --- | --- | --- | --- |
| **SNP** | **genotype** | **N^a^** | **median (IQR)** |
| *FUT2*  rs492602 | TT | 64 | 224 (168, 318) |
|  | TC | 143 | 290 (207, 399) |
|  | CC | 79 | 368 (239, 503) |
|  | ratio of geometric means per C allele  (95% CI) | 286 | 1.24  (1.13, 1.35) |
|  | p-value |  | 1.79x10^-6^ |
| *TCN2*  rs1801198 | GG | 53 | 284 (197, 423) |
|  | CG | 139 | 277 (193, 417) |
|  | CC | 91 | 287 (207, 393) |
|  | ratio of geometric means per C allele  (95% CI) | 283 | 1.00  (0.92-1.01) |
|  | p-value |  | 0.96 |
| *TCN2*  rs9606756 | AA | 232 | 301 (204, 420) |
|  | AG | 59 | 273 (196, 394) |
|  | GG | 6 | 223 (108, 227) |
|  | ratio of geometric means per G allele  (95% CI) | 297 | 0.88  (0.78, 1.01) |
|  | p-value |  | 0.06 |

^a^N reflects the number of children with genotype and cord blood vitamin B-12 data, regardless of whether there was IQ data available for them as well.
