# Supplementary material for: Vitamin B-12 Status during Pregnancy and Child’s IQ at Age 8: A Mendelian Randomization Study in the Avon Longitudinal Study of Parents and Children
Source: PLoS One. 2012 Dec 5;7(12):e51084. doi: 10.1371/journal.pone.0051084 (PMC3515553; doi:10.1371/journal.pone.0051084)
Supplement: Table S4 — Multivariable association of maternal genotype with offspring IQ at age 8. (DOCX) [file pone.0051084.s004.docx]

**Table S4.** Multivariable association of maternal genotype with offspring IQ at age 8. N=2299.

| **SNP** | **unadjusted** | **adjusted for child’s genotype** | **adjusted for child’s genotype and population stratification** |
| --- | --- | --- | --- |
| **rs492602** |  |  |  |
| mean difference in child IQ per C allele (95% CI) | 0.7  (-0.2, 1.6) | 0.9  (-0.2, 1.9) | 0.8  (-0.2, 1.9) |
| p-value | 0.13 | 0.11 | 0.12 |
| **rs1801198** |  |  |  |
| mean difference in child IQ per C allele (95% CI) | 0.4  (-0.5, 1.4) | 0.5  (-0.8, 1.5) | 0.3  (-0.8, 1.4) |
| p-value | 0.37 | 0.53 | 0.56 |
| **rs9606756** |  |  |  |
| mean difference in child IQ per G allele  (95% CI) | 0.5  (-1.0, 1.9) | 0.8  (-0.8, 2.5) | 0.8  (-0.9, 2.4) |
| p-value | 0.54 | 0.34 | 0.37 |
