# Supplementary material for: Vitamin B-12 Status during Pregnancy and Child’s IQ at Age 8: A Mendelian Randomization Study in the Avon Longitudinal Study of Parents and Children
Source: PLoS One. 2012 Dec 5;7(12):e51084. doi: 10.1371/journal.pone.0051084 (PMC3515553; doi:10.1371/journal.pone.0051084)
Supplement: Table S5 — Association of maternal genotype at SNPs related to vitamin B-12 metabolism with offspring IQ at age 8, stratified by maternal pregnancy recommended daily amount (RDA) of vitamin B-12. (DOCX) [file pone.0051084.s005.docx]

**Table S5.** Association of maternal genotype at SNPs related to vitamin B-12 metabolism with offspring IQ at age 8, stratified by maternal pregnancy recommended daily amount (RDA) of vitamin B-12 .

| **SNP** | **genotype** | **N** | **< RDA** | **N** | **≥ RDA** | **p-value for interaction** |
| --- | --- | --- | --- | --- | --- | --- |
|  |  |  | **mean IQ (SD)** |  | **mean IQ (SD)** |  |
| *FUT2*  rs492602 | TT | 123 | 98.7 (17.6) | 848 | 104.1 (16.7) | 0.51 |
|  | TC | 264 | 101.4 (15.1) | 1588 | 105.0 (16.1) |  |
|  | CC | 150 | 101.0 (17.8) | 825 | 106.1 (16.3) |  |
|  | mean difference in child IQ per C allele (95% CI) | 537 | 1.1  (-0.9, 3.1) | 3261 | 1.0  (0.2, 1.8) |  |
|  | p-value |  | 0.30 |  | 0.01 |  |
| *TCN2*  rs1801198 | GG | 112 | 101.3 (17.6) | 634 | 104.4 (16.4) | 0.71 |
|  | CG | 262 | 100.3 (15.9) | 1632 | 105.0 (15.9) |  |
|  | CC | 162 | 101.2 (17.2) | 997 | 105.5 (16.8) |  |
|  | mean difference in child IQ per C allele (95% CI) | 536 | 0.03  (-2.0, 2.0) | 3263 | 0.5  (-0.3, 1.3) |  |
|  | p-value |  | 0.98 |  | 0.21 |  |
| *TCN2*  rs9606756 | AA | 483 | 101.5 (16.5) | 3052 | 105.2 (15.8) | 0.63 |
|  | AG | 134 | 101.0 (16.7) | 871 | 105.6 (17.0) |  |
|  | GG | 12 | 108.3 (9.2) | 57 | 107.9 (15.0) |  |
|  | mean difference in child IQ per G allele (95% CI) | 629 | 0.6  (-2.1, 3.3) | 3980 | 0.6  (-0.5, 1.6) |  |
|  | p-value |  | 0.65 |  | 0.31 |  |
