# Supplementary material for: Vitamin B-12 Status during Pregnancy and Child’s IQ at Age 8: A Mendelian Randomization Study in the Avon Longitudinal Study of Parents and Children
Source: PLoS One. 2012 Dec 5;7(12):e51084. doi: 10.1371/journal.pone.0051084 (PMC3515553; doi:10.1371/journal.pone.0051084)
Supplement: Table S6 — Association of offspring IQ with potential covariables. (DOCX) [file pone.0051084.s006.docx]

**Table S6.** Association of offspring IQ with potential covariables.

|  |  | **Offspring IQ** | |  |
| --- | --- | --- | --- | --- |
|  | **N** | **mean** | **SD** | **p-value** |
| **Education** | 6159 |  |  | < 0.001 |
| < O level | 1327 | 96.4 | 15.0 |  |
| O level | 2163 | 102.4 | 15.0 |  |
| > O level | 2669 | 110.4 | 15.8 |  |
| **Social class** | 5357 |  |  | < 0.001 |
| manual | 851 | 98.8 | 15.3 |  |
| non-manual | 4506 | 106.4 | 16 |  |
| **Parity** | 6121 |  |  | < 0.001 |
| 0 children | 2852 | 105.9 | 16.2 |  |
| 1 child | 2195 | 104.6 | 16.2 |  |
| 2 children | 803 | 101.1 | 16.6 |  |
| ≥ 3 children | 271 | 99.1 | 15.3 |  |
| **Infection in pregnancy** | 6001 |  |  | 0.005 |
| no | 4769 | 105 | 16.4 |  |
| yes | 1232 | 103.5 | 16 |  |
| **Ever smoked** | 6141 |  |  | < 0.001 |
| no | 3428 | 105.5 | 16.4 |  |
| yes | 2713 | 103.3 | 16.2 |  |
| **Alcohol before pregnancy** | 6152 |  |  | < 0.001 |
| never | 335 | 98.8 | 16.3 |  |
| < 1 glass per week | 2295 | 103.2 | 16 |  |
| ≥ 1 glass per week | 2784 | 105.5 | 16.3 |  |
| ≥ 1 glass per day | 738 | 107.5 | 16.7 |  |
| **Alcohol in 1-3 mo gestation** | 6144 |  |  | 0.004 |
| never | 2678 | 104 | 16.2 |  |
| < 1 glass per week | 2523 | 104.8 | 16.6 |  |
| ≥ 1 glass per week | 840 | 105.8 | 15.8 |  |
| ≥ 1 glass per day | 103 | 101.2 | 18.1 |  |
| **Folate supplementation** | 6235 |  |  | < 0.001 |
| no | 4334 | 104 | 16.4 |  |
| yes | 1901 | 105.5 | 16.2 |  |
| **Offspring sex** | 6259 |  |  | 0.78 |
| boy | 3120 | 104.5 | 17 |  |
| girl | 3139 | 104.4 | 15.7 |  |
| **Breastfeeding** | 5842 |  |  | < 0.001 |
| never | 1160 | 98.6 | 15.4 |  |
| < 3 mo | 1330 | 102.9 | 15.8 |  |
| 3-5 mo | 1035 | 105.3 | 16.2 |  |
| ≥ 6 mo | 2317 | 108.6 | 16.2 |  |
| **Maternal age at delivery: mean difference in IQ per year (95% CI)** | 6259 | 0.7 | 0.6, 0.7 | < 0.001 |
| **Offspring age at testing: mean difference in IQ per month (95%CI)** | 6259 | -0.8 | -0.9, -0.7 | < 0.001 |
| **Gestation: mean difference in IQ per 1SD (95%CI)** | 6259 | 0.2 | -0.2, 0.6 | 0.39 |
| **Birth-weight: mean difference in IQ per 1SD (95%CI)** | 6184 | 1.1 | 0.7, 1.6 | < 0.001 |
