# Supplementary material for: Vitamin B-12 Status during Pregnancy and Child’s IQ at Age 8: A Mendelian Randomization Study in the Avon Longitudinal Study of Parents and Children
Source: PLoS One. 2012 Dec 5;7(12):e51084. doi: 10.1371/journal.pone.0051084 (PMC3515553; doi:10.1371/journal.pone.0051084)
Supplement: Table S7 — Association between maternal vitamin B-12 daily intake and potential covariables. (DOCX) [file pone.0051084.s007.docx]

**Table S7.** Association between maternal vitamin B-12 daily intake and potential covariables.

|  |  | **Dietary vitamin B-12 intake (ug/day)** | |  |
| --- | --- | --- | --- | --- |
|  | **N** | **median** | **IQR** | **p-value** |
| **Education** | 10795 |  |  | < 0.001 |
| < O level | 3164 | 3.82 | 2.77, 5.41 |  |
| O level | 3802 | 4.27 | 3.10, 6.21 |  |
| > O level | 3829 | 4.72 | 3.37, 6.53 |  |
| **Social class** | 9120 |  |  | < 0.001 |
| manual | 1803 | 4.03 | 2.90, 5.82 |  |
| non-manual | 7317 | 4.38 | 3.17, 6.25 |  |
| **Parity** | 10548 |  |  | < 0.001 |
| 0 children | 4740 | 4.07 | 2.95, 5.90 |  |
| 1 child | 3756 | 4.41 | 3.21, 6.33 |  |
| 2 children | 1485 | 4.59 | 3.22, 6.58 |  |
| ≥ 3 children | 567 | 4.46 | 3.25, 6.46 |  |
| **Infection in pregnancy** | 9903 |  |  | 0.77 |
| no | 7727 | 4.29 | 3.10, 6.18 |  |
| yes | 2176 | 4.32 | 3.10, 6.22 |  |
| **Ever smoked** | 10590 |  |  | 0.18 |
| no | 5338 | 4.33 | 3.09, 6.23 |  |
| yes | 5252 | 4.24 | 3.08, 6.12 |  |
| **Alcohol before pregnancy** | 10603 |  |  | < 0.001 |
| never | 717 | 3.55 | 2.76, 5.24 |  |
| < 1 glass per week | 3994 | 4.13 | 2.99, 6.01 |  |
| ≥ 1 glass per week | 4684 | 4.44 | 3.24, 6.29 |  |
| ≥ 1 glass per day | 1208 | 4.53 | 3.27, 6.46 |  |
| **Alcohol in 1-3 mo gestation** | 10580 |  |  | < 0.001 |
| never | 4708 | 4.12 | 2.97, 6.05 |  |
| < 1 glass per week | 4196 | 4.37 | 3.18, 6.21 |  |
| ≥ 1 glass per week | 1495 | 4.49 | 3.24, 6.45 |  |
| ≥ 1 glass per day | 181 | 4.45 | 3.35, 6.15 |  |
| **Folate supplementation** | 10851 |  |  | 0.01 |
| no | 7894 | 4.23 | 3.04, 6.11 |  |
| yes | 2957 | 4.43 | 3.17, 6.30 |  |
| **Offspring sex** | 10832 |  |  | 0.20 |
| boy | 5571 | 4.28 | 3.11, 6.20 |  |
| girl | 5261 | 4.27 | 3.05, 6.15 |  |
| **Breastfeeding** | 9253 |  |  | < 0.001 |
| never | 2371 | 3.83 | 2.82, 5.60 |  |
| < 3 mo | 2118 | 4.20 | 3.03, 5.93 |  |
| 3-5 mo | 1540 | 4.53 | 3.30, 6.39 |  |
| ≥ 6 mo | 3224 | 4.67 | 3.39, 6.56 |  |
| **Maternal age at delivery: mean difference in vitamin B-12 intake per year (95% CI)^a^** | 10851 | 0.05 | 0.04, 0.07 | < 0.001 |
| **Offspring age at testing: mean difference in vitamin B-12 intake per month (95%CI) ^a^** | 6125 | -0.02 | -0.04, -0.005 | 0.001 |
| **Gestation: mean difference in vitamin B-12 intake per 1SD (95%CI) ^a^** | 10851 | 0.01 | -0.04, 0.06 | 0.73 |
| **Birth-weight: mean difference in vitamin B-12 intake per 1SD (95%CI) ^a^** | 10713 | 0.09 | 0.04, 0.15 | < 0.001 |

Dietary vitamin B-12 intake was log-transformed for all tests of association. ^a^Regression coefficients and 95% CI shown were obtained by running a linear regression with the untransformed dietary vitamin B-12 intake variable.
