# Supplementary material for: Vitamin B-12 Status during Pregnancy and Child’s IQ at Age 8: A Mendelian Randomization Study in the Avon Longitudinal Study of Parents and Children
Source: PLoS One. 2012 Dec 5;7(12):e51084. doi: 10.1371/journal.pone.0051084 (PMC3515553; doi:10.1371/journal.pone.0051084)
Supplement: Table S8 — Association between maternal genotype at rs492602 and potential covariables. (DOCX) [file pone.0051084.s008.docx]

**Table S8.** Association between maternal genotype at rs492602 and potential covariables.

|  |  | **% of each covariable category by genotype** | | |  |
| --- | --- | --- | --- | --- | --- |
|  | **N** | **TT** | **TC** | **CC** | **p-value** |
| **Education** | 6634 |  |  |  | 0.12 |
| < O level | 1885 | 30.1 | 28.1 | 27.2 |  |
| O level | 2359 | 36.1 | 35.7 | 34.8 |  |
| > O level | 2390 | 33.8 | 36.2 | 38.0 |  |
| **Social class** | 5520 |  |  |  | 0.14 |
| Manual | 1055 | 18.0 | 20.2 | 18.2 |  |
| Non-manual | 4465 | 82.0 | 79.8 | 81.8 |  |
| **Parity** | 6777 |  |  |  | 0.45 |
| no children | 3104 | 45.7 | 46.0 | 45.5 |  |
| 1 child | 2366 | 33.9 | 35.5 | 34.8 |  |
| 2 children | 934 | 14.9 | 13.4 | 13.4 |  |
| ≥ 3 children | 373 | 5.5 | 5.1 | 6.3 |  |
| **Infection in pregnancy** | 6354 |  |  |  | 0.03 |
| no | 4931 | 76.5 | 79.0 | 76.1 |  |
| yes | 1423 | 23.5 | 21.0 | 23.9 |  |
| **Ever smoked** | 6801 |  |  |  | 0.40 |
| no | 3444 | 50.2 | 50.2 | 52.1 |  |
| yes | 3357 | 49.8 | 49.8 | 47.9 |  |
| **Alcohol before pregnancy** | 6807 |  |  |  | 0.37 |
| never | 466 | 6.9 | 7.2 | 6.1 |  |
| < 1 glass per week | 2524 | 38.6 | 36.9 | 35.9 |  |
| ≥ 1 glass per week | 3038 | 42.8 | 44.7 | 46.3 |  |
| ≥ 1 glass per day | 779 | 11.7 | 11.2 | 11.7 |  |
| **Alcohol in 1-3 mo gestation** | 6791 |  |  |  | 0.49 |
| never | 3030 | 46.3 | 44.9 | 42.5 |  |
| < 1 glass per week | 2662 | 38.1 | 39.2 | 40.4 |  |
| ≥ 1 glass per week | 975 | 13.9 | 14.1 | 15.2 |  |
| ≥ 1 glass per day | 124 | 1.7 | 1.8 | 1.9 |  |
| **Folate supplementation** | 6973 |  |  |  | 0.31 |
| no | 4786 | 68.5 | 69.4 | 67.3 |  |
| yes | 2187 | 31.5 | 30.6 | 32.7 |  |
| **Offspring sex** | 7118 |  |  |  | 0.16 |
| boy | 3649 | 51.9 | 52.0 | 49.3 |  |
| girl | 3469 | 48.1 | 48.0 | 50.7 |  |
| **Breastfeeding** | 5932 |  |  |  | 0.41 |
| never | 1541 | 26.2 | 26.5 | 24.8 |  |
| < 3 mo | 1383 | 23.7 | 23.8 | 21.9 |  |
| 3-5 mo | 974 | 16.6 | 16.2 | 16.8 |  |
| ≥ 6 mo | 2034 | 33.5 | 33.5 | 36.5 |  |
| **Maternal age at delivery: mean (SD) (years)** | 7118 | 28.2 (4.8) | 28.2 (4.6) | 28.3 (4.9) | 0.49 |
| **Offspring age at testing: mean (SD) (mos)** | 4026 | 103.5 (3.3) | 103.5 (3.1) | 103.5 (3.2) | 0.93 |
| **Gestation: mean (SD) (weeks)** | 7118 | 39.5 (1.7) | 39.6 (1.7) | 39.6 (1.7) | 0.39 |
| **Birth-weight: mean (SD) (g)** | 7042 | 3417.1 (523.6) | 3441.8 (534.3) | 3422.0 (526.1) | 0.20 |
