# Supplementary material for: Vitamin B-12 Status during Pregnancy and Child’s IQ at Age 8: A Mendelian Randomization Study in the Avon Longitudinal Study of Parents and Children
Source: PLoS One. 2012 Dec 5;7(12):e51084. doi: 10.1371/journal.pone.0051084 (PMC3515553; doi:10.1371/journal.pone.0051084)
Supplement: Table S9 — Association between offspring genotype at rs492602 and potential covariables. (DOCX) [file pone.0051084.s009.docx]

**Table S9.** Association between offspring genotype at rs492602 and potential covariables.

|  |  | **% of each covariable category by genotype** | | |  |
| --- | --- | --- | --- | --- | --- |
|  | **N** | **TT** | **TC** | **CC** | **p-value** |
| **Education** | 7248 |  |  |  | 0.88 |
| < O level | 1901 | 26.3 | 26.3 | 26.0 |  |
| O level | 2562 | 36.0 | 35.3 | 34.7 |  |
| > O level | 2785 | 37.7 | 38.4 | 39.3 |  |
| **Social class** | 6085 |  |  |  | 0.55 |
| Manual | 1098 | 18.5 | 18.3 | 17.1 |  |
| Non-manual | 4987 | 81.5 | 81.7 | 82.9 |  |
| **Parity** | 7261 |  |  |  | 0.01 |
| no children | 3228 | 48.1 | 43.2 | 43.3 |  |
| 1 child | 2630 | 34.1 | 36.4 | 37.9 |  |
| 2 children | 1012 | 12.7 | 14.6 | 13.9 |  |
| ≥ 3 children | 391 | 5.1 | 5.8 | 4.9 |  |
| **Infection in pregnancy** | 6904 |  |  |  | 0.71 |
| no | 5435 | 78.5 | 78.5 | 79.4 |  |
| yes | 1469 | 21.5 | 21.5 | 20.6 |  |
| **Ever smoked** | 7285 |  |  |  | 0.09 |
| no | 3819 | 51.5 | 51.8 | 54.6 |  |
| yes | 3466 | 48.5 | 48.2 | 45.4 |  |
| **Alcohol before pregnancy** | 7283 |  |  |  | 0.03 |
| never | 451 | 7.3 | 5.9 | 5.7 |  |
| < 1 glass per week | 2722 | 37.1 | 37.4 | 37.6 |  |
| ≥ 1 glass per week | 3261 | 46.0 | 44.4 | 44.3 |  |
| ≥ 1 glass per day | 849 | 9.6 | 12.3 | 12.4 |  |
| **Alcohol in 1-3 mo gestation** | 7264 |  |  |  | 0.02 |
| never | 3168 | 46.0 | 42.7 | 43.0 |  |
| < 1 glass per week | 2915 | 39.2 | 41.2 | 39.0 |  |
| ≥ 1 glass per week | 1060 | 13.7 | 14.2 | 16.2 |  |
| ≥ 1 glass per day | 121 | 1.1 | 1.9 | 1.8 |  |
| **Folate supplementation** | 7445 |  |  |  | 0.87 |
| no | 5264 | 70.3 | 71.0 | 70.6 |  |
| yes | 2181 | 29.7 | 29.0 | 29.4 |  |
| **Offspring sex** | 7524 |  |  |  | 0.13 |
| boy | 3923 | 51.7 | 51.4 | 54.1 |  |
| girl | 3601 | 48.3 | 48.6 | 45.9 |  |
| **Breastfeeding** | 6548 |  |  |  | 0.44 |
| never | 1565 | 23.2 | 24.9 | 22.7 |  |
| < 3 mo | 1477 | 23.6 | 22.1 | 22.4 |  |
| 3-5 mo | 1124 | 17.8 | 16.9 | 17.1 |  |
| ≥ 6 mo | 2382 | 35.4 | 36.1 | 37.9 |  |
| **Maternal age at delivery: mean (SD) (years)** | 7524 | 28.4 (4.7) | 28.7 (4.8) | 28.6 (4.7) | 0.11 |
| **Offspring age at testing: mean (SD) (mos)** | 5010 | 103.3 (3.3) | 103.3 (3.0) | 103.2 (2.9) | 0.89 |
| **Gestation: mean (SD) (weeks)** | 7524 | 39.5 (1.7) | 39.6 (1.7) | 39.6 (1.6) | 0.24 |
| **Birth-weight: mean (SD) (g)** | 7434 | 3439.7 (523.8) | 3464.9 (515.0) | 3450.1 (525.6) | 0.21 |
