# Supplementary material for: Vitamin B-12 Status during Pregnancy and Child’s IQ at Age 8: A Mendelian Randomization Study in the Avon Longitudinal Study of Parents and Children
Source: PLoS One. 2012 Dec 5;7(12):e51084. doi: 10.1371/journal.pone.0051084 (PMC3515553; doi:10.1371/journal.pone.0051084)
Supplement: Table S10 — Association between maternal genotype at rs1801198 and potential covariables. (DOCX) [file pone.0051084.s010.docx]

**Table S10.** Association between maternal genotype at rs1801198 and potential covariables.

|  |  | **% of each covariable category by genotype** | | |  |
| --- | --- | --- | --- | --- | --- |
|  | **N** | **GG** | **GC** | **CC** | **p-value** |
| **Education** | 6663 |  |  |  | 0.38 |
| < O level | 1906 | 28.5 | 27.8 | 30.0 |  |
| O level | 2371 | 34.8 | 35.9 | 35.6 |  |
| > O level | 2386 | 36.8 | 36.3 | 34.4 |  |
| **Social class** | 5532 |  |  |  | 0.40 |
| Manual | 1063 | 19.5 | 18.5 | 20.1 |  |
| Non-manual | 4469 | 80.5 | 81.5 | 79.9 |  |
| **Parity** | 6811 |  |  |  | 0.06 |
| no children | 3118 | 45.9 | 44.6 | 47.6 |  |
| 1 child | 2374 | 35.3 | 35.4 | 33.7 |  |
| 2 children | 941 | 12.2 | 14.5 | 13.8 |  |
| ≥ 3 children | 378 | 6.7 | 5.5 | 4.9 |  |
| **Infection in pregnancy** | 6381 |  |  |  | 0.86 |
| no | 4945 | 77.3 | 77.3 | 77.9 |  |
| yes | 1436 | 22.7 | 22.7 | 22.1 |  |
| **Ever smoked** | 6837 |  |  |  | 0.37 |
| no | 3447 | 50.8 | 51.1 | 49.1 |  |
| yes | 3390 | 49.2 | 48.9 | 50.9 |  |
| **Alcohol before pregnancy** | 6843 |  |  |  | 0.63 |
| never | 474 | 6.8 | 7.0 | 7.0 |  |
| < 1 glass per week | 2533 | 36.7 | 37.9 | 35.8 |  |
| ≥ 1 glass per week | 3050 | 45.3 | 44.2 | 44.8 |  |
| ≥ 1 glass per day | 786 | 11.4 | 11.0 | 12.4 |  |
| **Alcohol in 1-3 mo gestation** | 6827 |  |  |  | 0.27 |
| never | 3051 | 43.8 | 44.4 | 45.7 |  |
| < 1 glass per week | 2663 | 41.2 | 39.1 | 37.5 |  |
| ≥ 1 glass per week | 983 | 13.7 | 14.6 | 14.6 |  |
| ≥ 1 glass per day | 130 | 1.3 | 1.9 | 2.2 |  |
| **Folate supplementation** | 7008 |  |  |  | 0.46 |
| no | 4811 | 67.7 | 69.3 | 68.2 |  |
| yes | 2197 | 32.3 | 30.7 | 31.8 |  |
| **Offspring sex** | 7154 |  |  |  | 0.28 |
| boy | 3675 | 53.2 | 50.7 | 51.3 |  |
| girl | 3479 | 46.8 | 49.3 | 48.7 |  |
| **Breastfeeding** | 5952 |  |  |  | 0.13 |
| never | 1546 | 26.2 | 24.8 | 27.7 |  |
| < 3 mo | 1385 | 25.2 | 23.4 | 21.8 |  |
| 3-5 mo | 978 | 16.6 | 16.8 | 15.8 |  |
| ≥ 6 mo | 2043 | 32.1 | 35.0 | 34.7 |  |
| **Maternal age at delivery: mean (SD) (years)** | 7154 | 28.2 (4.8) | 28.4 (4.7) | 28.1 (4.9) | 0.07 |
| **Offspring age at testing: mean (SD) (mos)** | 4039 | 103.3 (3.1) | 103.5 (3.3) | 103.5 (3.1) | 0.35 |
| **Gestation: mean (SD) (weeks)** | 7154 | 39.6 (1.7) | 39.5 (1.7) | 39.5 (1.8) | 0.11 |
| **Birth-weight: mean (SD) (g)** | 7077 | 3432.4 (530.2) | 3432.3 (522.4) | 3430.1 (542.2) | 0.99 |
