# Supplementary material for: Vitamin B-12 Status during Pregnancy and Child’s IQ at Age 8: A Mendelian Randomization Study in the Avon Longitudinal Study of Parents and Children
Source: PLoS One. 2012 Dec 5;7(12):e51084. doi: 10.1371/journal.pone.0051084 (PMC3515553; doi:10.1371/journal.pone.0051084)
Supplement: Table S11 — Association between offspring genotype at rs1801198 and potential covariables. (DOCX) [file pone.0051084.s011.docx]

**Table S11.** Association between offspring genotype at rs1801198 and potential covariables.

|  |  | **% of each covariable category by genotype** | | |  |
| --- | --- | --- | --- | --- | --- |
|  | **N** | **GG** | **GC** | **CC** | **p-value** |
| **Education** | 7254 |  |  |  | 0.80 |
| < O level | 1909 | 26.8 | 25.9 | 26.7 |  |
| O level | 2568 | 35.3 | 36.0 | 34.4 |  |
| > O level | 2777 | 37.9 | 38.1 | 38.9 |  |
| **Social class** | 6096 |  |  |  | 0.92 |
| Manual | 1114 | 18.6 | 18.3 | 18.0 |  |
| Non-manual | 4982 | 81.4 | 81.7 | 82.0 |  |
| **Parity** | 7269 |  |  |  | 0.16 |
| no children | 3230 | 42.4 | 45.0 | 44.7 |  |
| 1 child | 2635 | 38.3 | 34.9 | 37.2 |  |
| 2 children | 1018 | 14.0 | 14.4 | 13.4 |  |
| ≥ 3 children | 386 | 5.3 | 5.7 | 4.7 |  |
| **Infection in pregnancy** | 6910 |  |  |  | 0.87 |
| no | 5439 | 78.2 | 78.8 | 78.9 |  |
| yes | 1471 | 21.8 | 21.2 | 21.1 |  |
| **Ever smoked** | 7291 |  |  |  | 0.28 |
| no | 3817 | 50.9 | 53.2 | 51.8 |  |
| yes | 3474 | 49.1 | 46.8 | 48.2 |  |
| **Alcohol before pregnancy** | 7290 |  |  |  | 0.93 |
| never | 459 | 6.7 | 6.1 | 6.4 |  |
| < 1 glass per week | 2728 | 37.4 | 37.7 | 36.9 |  |
| ≥ 1 glass per week | 3264 | 44.6 | 44.4 | 45.5 |  |
| ≥ 1 glass per day | 839 | 11.4 | 11.8 | 11.2 |  |
| **Alcohol in 1-3 mo gestation** | 7271 |  |  |  | 0.17 |
| never | 3175 | 43.2 | 43.3 | 44.5 |  |
| < 1 glass per week | 2923 | 42.5 | 40.4 | 38.4 |  |
| ≥ 1 glass per week | 1054 | 12.8 | 14.7 | 15.3 |  |
| ≥ 1 glass per day | 119 | 1.5 | 1.6 | 1.8 |  |
| **Folate supplementation** | 7449 |  |  |  | 0.16 |
| no | 5267 | 69.1 | 70.6 | 72.0 |  |
| yes | 2182 | 30.9 | 29.4 | 28.0 |  |
| **Offspring sex** | 7527 |  |  |  | 0.41 |
| boy | 3897 | 50.7 | 51.6 | 52.8 |  |
| girl | 3630 | 49.3 | 48.4 | 47.2 |  |
| **Breastfeeding** | 6559 |  |  |  | 0.31 |
| never | 1568 | 22.3 | 23.9 | 25.0 |  |
| < 3 mo | 1470 | 22.7 | 23.1 | 21.2 |  |
| 3-5 mo | 1133 | 18.9 | 16.6 | 17.3 |  |
| ≥ 6 mo | 2388 | 36.1 | 36.4 | 36.5 |  |
| **Maternal age at delivery: mean (SD) (years)** | 7527 | 28.5 (4.6) | 28.6 (4.8) | 28.6 (4.8) | 0.83 |
| **Offspring age at testing: mean (SD) (mos)** | 5004 | 103.4 (3.2) | 103.3 (3.0) | 103.2 (3.0) | 0.32 |
| **Gestation: mean (SD) (weeks)** | 7527 | 39.6 (1.7) | 39.6 (1.6) | 39.5 (1.8) | 0.11 |
| **Birth-weight: mean (SD)(g)** | 7438 | 3471.0 (518.0) | 3445.0 (509.4) | 3461.3 (535.1) | 0.21 |
