# Supplementary material for: Vitamin B-12 Status during Pregnancy and Child’s IQ at Age 8: A Mendelian Randomization Study in the Avon Longitudinal Study of Parents and Children
Source: PLoS One. 2012 Dec 5;7(12):e51084. doi: 10.1371/journal.pone.0051084 (PMC3515553; doi:10.1371/journal.pone.0051084)
Supplement: Table S12 — Association between maternal genotype at rs9606756 and potential covariables. (DOCX) [file pone.0051084.s012.docx]

**Table S12.** Association between maternal genotype at rs9606756 and potential covariables.

|  |  | **% of each covariable category by genotype** | | |  |
| --- | --- | --- | --- | --- | --- |
|  | **N** | **AA** | **AG** | **GG** | **p-value** |
| **Education** | 7433 |  |  |  | 0.60 |
| < O level | 1990 | 26.6 | 27.5 | 26.2 |  |
| O level | 2632 | 35.2 | 35.8 | 40.2 |  |
| > O level | 2811 | 38.2 | 36.7 | 33.6 |  |
| **Social class** | 6253 |  |  |  | 0.24 |
| Manual | 1134 | 17.8 | 18.8 | 23.6 |  |
| Non-manual | 5119 | 82.2 | 81.2 | 76.4 |  |
| **Parity** | 7515 |  |  |  | 0.01 |
| no children | 3469 | 45.4 | 48.3 | 49.6 |  |
| 1 child | 2634 | 35.2 | 34.8 | 30.1 |  |
| 2 children | 1042 | 14.3 | 12.6 | 9.8 |  |
| ≥ 3 children | 370 | 5.0 | 4.3 | 10.5 |  |
| **Infection in pregnancy** | 7079 |  |  |  | 0.95 |
| no | 5532 | 78.2 | 78.0 | 77.1 |  |
| yes | 1547 | 21.8 | 22.0 | 22.9 |  |
| **Ever smoked** | 7545 |  |  |  | 0.37 |
| no | 3900 | 51.4 | 52.4 | 56.9 |  |
| yes | 3645 | 48.6 | 47.6 | 43.1 |  |
| **Alcohol before pregnancy** | 7555 |  |  |  | 0.40 |
| never | 507 | 6.4 | 7.6 | 9.8 |  |
| < 1 glass per week | 2797 | 36.9 | 37.2 | 38.2 |  |
| ≥ 1 glass per week | 3362 | 44.8 | 43.7 | 43.1 |  |
| ≥ 1 glass per day | 889 | 11.9 | 11.5 | 8.9 |  |
| **Alcohol in 1-3 mo gestation** | 7536 |  |  |  | 0.54 |
| never | 3336 | 43.8 | 45.3 | 52.0 |  |
| < 1 glass per week | 3005 | 40.2 | 38.9 | 36.6 |  |
| ≥ 1 glass per week | 1069 | 14.3 | 14.1 | 10.6 |  |
| ≥ 1 glass per day | 126 | 1.7 | 1.7 | 0.8 |  |
| **Folate supplementation** | 7720 |  |  |  | 0.60 |
| no | 5399 | 69.6 | 70.9 | 70.3 |  |
| yes | 2321 | 30.4 | 29.1 | 29.7 |  |
| **Offspring sex** | 7831 |  |  |  | 0.97 |
| boy | 3939 | 50.4 | 50.1 | 49.6 |  |
| girl | 3892 | 49.6 | 49.9 | 50.4 |  |
| **Breastfeeding** | 6672 |  |  |  | 0.28 |
| never | 1616 | 23.7 | 25.5 | 31.6 |  |
| < 3 mo | 1498 | 23.0 | 20.9 | 20.2 |  |
| 3-5 mo | 1123 | 16.9 | 16.6 | 16.6 |  |
| ≥ 6 mo | 2435 | 36.4 | 37.0 | 31.6 |  |
| **Maternal age at delivery: mean (SD) (years)** | 7832 | 28.4 (4.7) | 28.3 (4.9) | 28.3 (4.6) | 0.60 |
| **Offspring age at testing: mean (SD) (mos)** | 4864 | 103.3 (3.1) | 103.5 (3.3) | 103.2 (3.7) | 0.11 |
| **Gestation: mean (SD) (weeks)** | 7832 | 39.6 (1.7) | 39.6 (1.7) | 39.4 (1.8) | 0.53 |
| **Birth-weight: mean (SD)(g)** | 7732 | 3428.7 (528.5) | 3433.1 (523.8) | 3483.4 (506.9) | 0.50 |
