# Supplementary material for: Vitamin B-12 Status during Pregnancy and Child’s IQ at Age 8: A Mendelian Randomization Study in the Avon Longitudinal Study of Parents and Children
Source: PLoS One. 2012 Dec 5;7(12):e51084. doi: 10.1371/journal.pone.0051084 (PMC3515553; doi:10.1371/journal.pone.0051084)
Supplement: Table S13 — Association between offspring genotype at rs9606756 and potential covariables. (DOCX) [file pone.0051084.s013.docx]

**Table S13.** Association between offspring genotype at rs9606756 and potential covariables.

|  |  | **% of each covariable category by genotype** | | |  |
| --- | --- | --- | --- | --- | --- |
|  | **N** | **AA** | **AG** | **GG** | **p-value** |
| **Education** | 7658 |  |  |  | 0.29 |
| < O level | 1976 | 25.7 | 26.8 | 19.6 |  |
| O level | 2697 | 35.1 | 35.0 | 42.0 |  |
| > O level | 2985 | 39.2 | 38.2 | 38.4 |  |
| **Social class** | 6473 |  |  |  | 0.14 |
| Manual | 1165 | 17.6 | 18.8 | 23.8 |  |
| Non-manual | 5308 | 82.4 | 81.2 | 76.2 |  |
| **Parity** | 7666 |  |  |  | 0.66 |
| no children | 3422 | 44.5 | 45.5 | 40.9 |  |
| 1 child | 2791 | 36.2 | 36.9 | 38.7 |  |
| 2 children | 1059 | 14.1 | 12.6 | 16.0 |  |
| ≥ 3 children | 394 | 5.2 | 5.0 | 4.4 |  |
| **Infection in pregnancy** | 7312 |  |  |  | 0.56 |
| no | 5747 | 78.7 | 78.4 | 74.8 |  |
| yes | 1565 | 21.3 | 21.6 | 25.2 |  |
| **Ever smoked** | 7693 |  |  |  | 0.62 |
| no | 4042 | 52.4 | 52.8 | 56.5 |  |
| yes | 3651 | 47.6 | 47.2 | 43.5 |  |
| **Alcohol before pregnancy** | 7695 |  |  |  | 0.96 |
| never | 469 | 6.0 | 6.4 | 7.3 |  |
| < 1 glass per week | 2879 | 37.6 | 36.7 | 36.2 |  |
| ≥ 1 glass per week | 3440 | 44.7 | 44.7 | 45.6 |  |
| ≥ 1 glass per day | 907 | 11.7 | 12.2 | 10.9 |  |
| **Alcohol in 1-3 mo gestation** | 7679 |  |  |  | 0.19 |
| never | 3347 | 43.3 | 44.2 | 46.8 |  |
| < 1 glass per week | 3114 | 40.8 | 40.5 | 30.9 |  |
| ≥ 1 glass per week | 1089 | 14.2 | 13.6 | 20.9 |  |
| ≥ 1 glass per day | 129 | 1.7 | 1.7 | 1.4 |  |
| **Folate supplementation** | 7846 |  |  |  | 0.21 |
| no | 5523 | 70.2 | 71.5 | 65.0 |  |
| yes | 2323 | 29.8 | 28.5 | 35.0 |  |
| **Offspring sex** | 7917 |  |  |  | 0.11 |
| boy | 4069 | 51.2 | 52.7 | 43.9 |  |
| girl | 3848 | 48.8 | 47.3 | 56.1 |  |
| **Breastfeeding** | 6964 |  |  |  | 0.07 |
| never | 1610 | 22.3 | 25.9 | 22.9 |  |
| < 3 mo | 1562 | 22.7 | 21.3 | 25.2 |  |
| 3-5 mo | 1208 | 17.8 | 15.7 | 19.1 |  |
| ≥ 6 mo | 2584 | 37.2 | 37.1 | 32.8 |  |
| **Maternal age at delivery: mean (SD) (years)** | 7917 | 28.6 (4.7) | 28.4 (4.8) | 29.2 (5.0) | 0.05 |
| **Offspring age at testing: mean (SD) (mos)** | 5487 | 103.3 (3.2) | 103.4 (3.3) | 103.3 (3.0) | 0.88 |
| **Gestation: mean (SD) (weeks)** | 7917 | 39.6 (1.7) | 39.6 (1.7) | 39.5 (2.1) | 0.93 |
| **Birth-weight: mean (SD)(g)** | 7824 | 3452.8 (521.6) | 3438.5 (509.9) | 3526.8 (568.9) | 0.14 |
